# Supplementary figures and images for: Dynamic modeling of EEG responses to natural speech reveals earlier processing of predictable words
Source: PLoS Comput Biol. 2025 Apr 28;21(4):e1013006. doi: 10.1371/journal.pcbi.1013006 (PMC12061398; doi:10.1371/journal.pcbi.1013006)

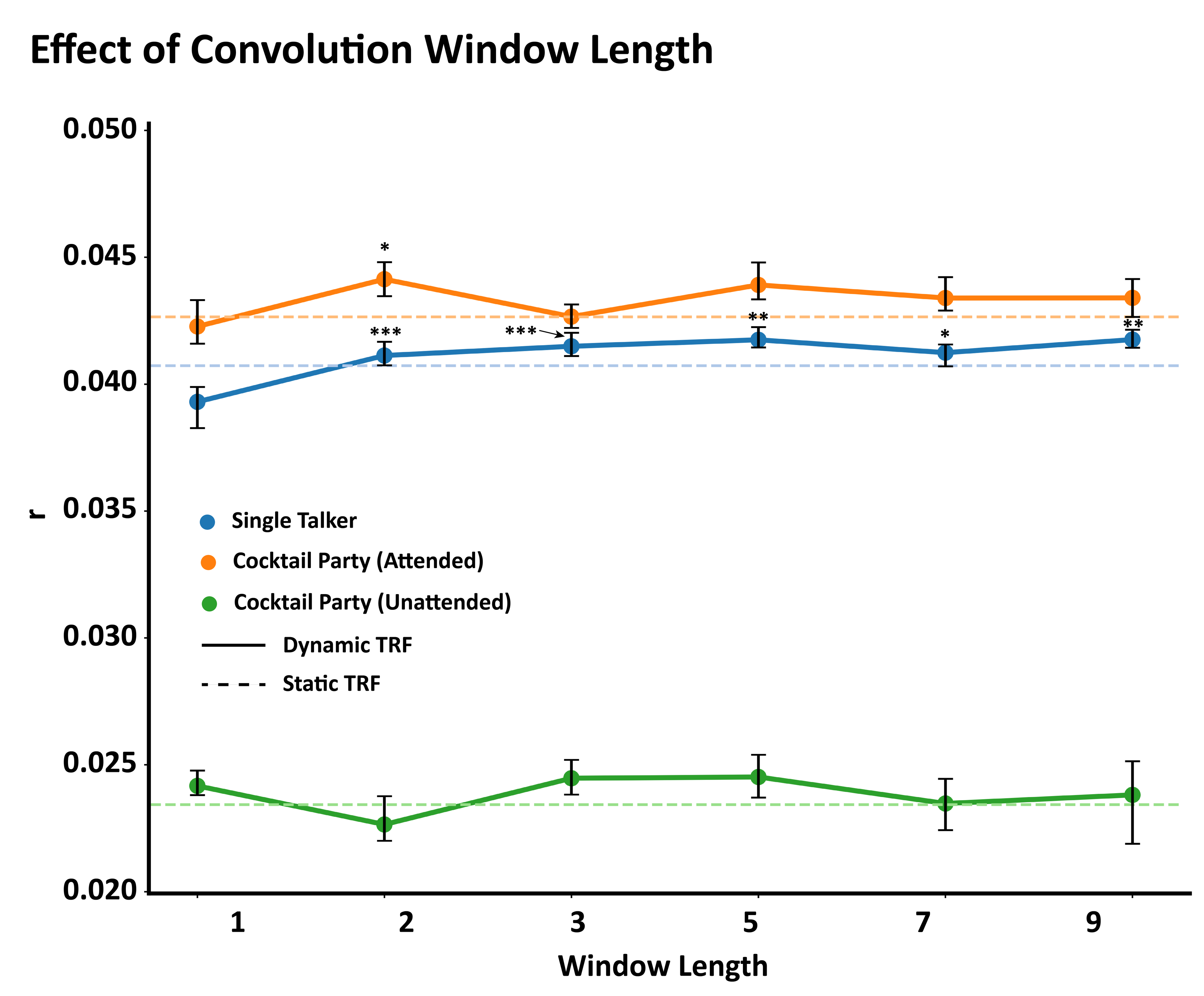

Supplement: S1 Fig — This figure plots the prediction accuracy (the median across subjects) for the dynamic vs. the fixed TRF models as a function of the window length used to predict the amplitude scaling and time-shift parameters of the variable TRF. Results are shown separately for the single talker, attended and unattended conditions. The dashed line indicates the performance for the static TRF. For single talker and attended speech, the model achieved the highest prediction accuracy when its window size was around 2–3 (the improvement from 2 to 3 for single talker was not significant, W = 133.0, p = 0.0668, single-tail). For unattended speech, the prediction accuracy was always not significant compared with the accuracy of the static TRF. (TIF) [file pcbi.1013006.s001.tif]

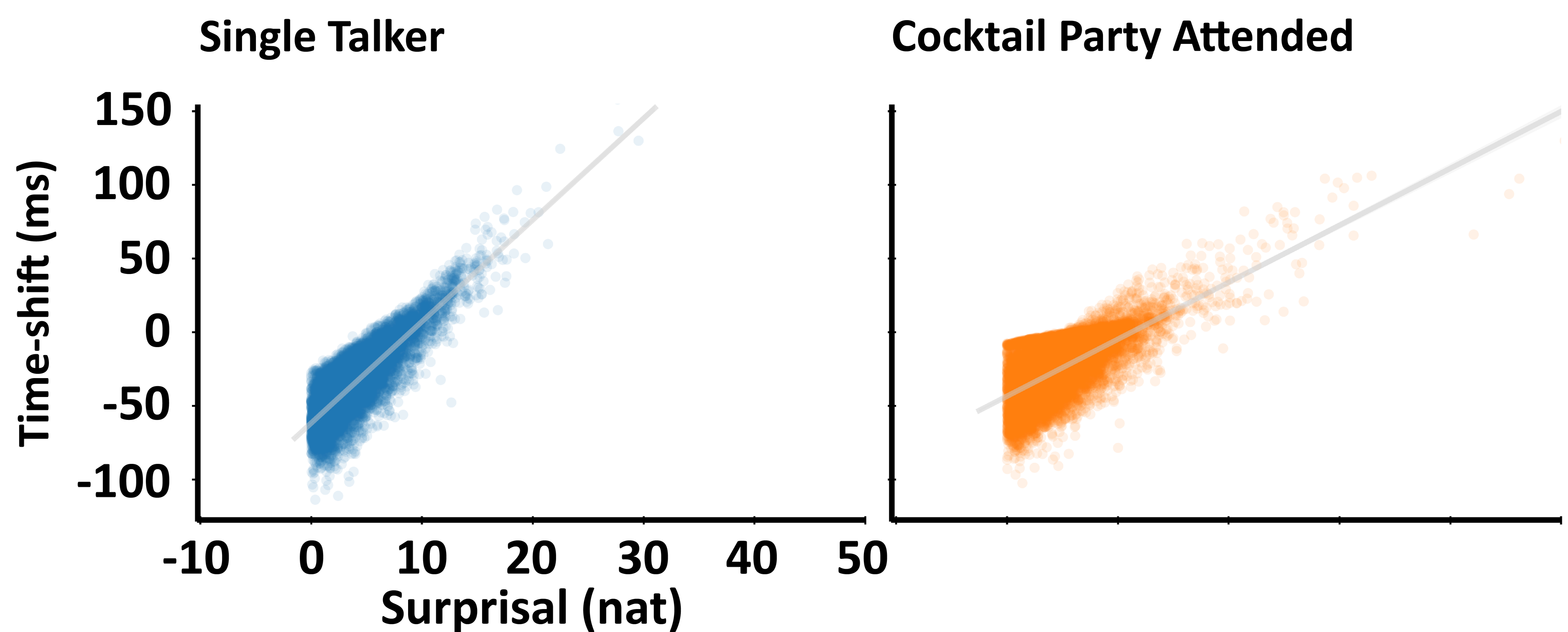

Supplement: S2 Fig — The scatterplot for surprisal and the time-shift. Each point indicates a pair of these two variables of a word. (TIF) [file pcbi.1013006.s002.tif]
