# Supplementary material for: Dynamic modeling of EEG responses to natural speech reveals earlier processing of predictable words
Source: PLoS Comput Biol. 2025 Apr 28;21(4):e1013006. doi: 10.1371/journal.pcbi.1013006 (PMC12061398; doi:10.1371/journal.pcbi.1013006)
Supplement: S1 Text — (DOCX) [file pcbi.1013006.s003.docx]

**Influence of Convolutional Window Length**

In the analyses presented in the main body of the manuscript, we used a convolutional window of two when fitting all dynamic TRFs. This means that the causal convolution layer that learns the TRF amplitude, time-shifting and time-scaling parameters only uses the lexical surprisal values for the current word and the previous word. To validate that this was a reasonable choice, we tested if using other window lengths would improve the ability of the dynamic TRF to predict left out EEG data. We did this based on the Time&Amp dynamic TRF as this was generally the best performing model across our various datasets.

The extended figure (S1 Fig) shows the median prediction accuracy (averaged across all scalp channels) for the static TRF (dashed lines), and for the Time&Amp dynamic TRF (dots, solid lines). The error bars show the central 68% of the sampling distribution (equivalent to one standard error for a Gaussian). Error bars were calculated using within-subject standard errors [1], which removes variation across subjects that is shared between all conditions. Specifically, before calculating the central interval, the mean for each subject across all conditions was removed. A correction factor was added to the interval to make the estimation unbiased. The correction factor was calculated as $\sqrt{N/N-1}$, where $N$ indicates the number of conditions. In the single talker condition, improvements started to be significant for a time window of two, however, the improvement in prediction accuracy for a window length of 3 over 2 was not significant (p = 0.0668, Wilcoxon Signed-rank Test). In the attended speech condition, only the time window of 2 had significant improvement over the fixed TRF. In the unattended speech condition, there was no significant improvement for any window length. These results suggest that the lexical surprisal for the current and previous words are the most influential in modulating the amplitude and time latency of the N400 TRF.

1. Loftus GR, Masson MEJ. Using confidence intervals in within-subject designs. Psychon Bull Rev. 1994;1: 476–490. doi:10.3758/BF03210951
