# Supplementary material for: Dynamic modeling of EEG responses to natural speech reveals earlier processing of predictable words
Source: PLoS Comput Biol. 2025 Apr 28;21(4):e1013006. doi: 10.1371/journal.pcbi.1013006 (PMC12061398; doi:10.1371/journal.pcbi.1013006)
Supplement: S2 Text — (DOCX) [file pcbi.1013006.s004.docx]

Incidentally, it is worth noting here that some previous work has fit TRFs using the semantic dissimilarity of words instead of their context-based lexical surprisal [1–3]. Semantic dissimilarity in those studies was defined using models that do not take into account the context in which a word is heard – they are known as bag-of-words models. With the arrival of transformer models like GPT (which are trained on next word (really token) prediction), it has become possible to derive more direct quantitative measures of how predictable a word is based on its context. Given our wish to compare our findings with the classic N400 and our overarching hypothesis – that responses to individual words may be processed more rapidly when they are predictable – we use lexical surprisal in the present study. Exploring how semantic dissimilarity might influence the timing of responses is a question that we think is worthy of future study.

1. Broderick MP, Anderson AJ, Di Liberto GM, Crosse MJ, Lalor EC. Electrophysiological correlates of semantic dissimilarity reflect the comprehension of natural, narrative speech. Current Biology. 2018;28: 803–809.

2. Broderick MP, Anderson AJ, Lalor EC. Semantic Context Enhances the Early Auditory Encoding of Natural Speech. J Neurosci. 2019;39: 7564–7575. doi:10.1523/JNEUROSCI.0584-19.2019

3. Broderick MP, Di Liberto GM, Anderson AJ, Rofes A, Lalor EC. Dissociable electrophysiological measures of natural language processing reveal differences in speech comprehension strategy in healthy ageing. Scientific reports. 2021;11: 4963.
