# Supplementary material for: Dynamic modeling of EEG responses to natural speech reveals earlier processing of predictable words
Source: PLoS Comput Biol. 2025 Apr 28;21(4):e1013006. doi: 10.1371/journal.pcbi.1013006 (PMC12061398; doi:10.1371/journal.pcbi.1013006)
Supplement: Attachment — Submitted filename: Response to reviewers.pdf [file pcbi.1013006.s007.pdf]

Many thanks to the reviewers and editors for their efforts in providing us with feedback on our manuscript. We greatly appreciate the time and effort taken. And we believe that the comments have been very useful in helping us to improve our manuscript.

While we respond to the individual comments point-by-point below, we would first like to highlight the most substantial changes. These include:

- Adding more context on the debate around the latency of the N400 to the introduction to help to better frame the goals and contribution of our study.
- Attempting to better explain – and to better justify – the specific (admittedly complex) approach we have used in the study.

We would also like to note another change that we made to our analysis in the revision that was not specifically prompted by any of the reviewer comments. It relates to the way in which we align the timing of the static and dynamic TRFs. Specifically, static TRFs are chosen to have a fixed duration – which corresponds to the convolutional window – in our case, we chose that to be 0 – 700 ms to ensure we were capturing all relevant brain activity to the words. However, the dynamic TRF can be shifted or stretched – it can move/stretch + or – 200ms at either end. In our initial submission, we had modified the dynamic TRF while fixing -200 ms as an anchor point. However, on reflection, this complicated comparison with the static TRF. As such, in our revision, we also changed how we modified the dynamic TRF making 0 ms be the anchor point to allow for more direct comparison with the static TRF. The specific numbers on some of our statistical tests changed slightly, but there was no qualitative change to the results. We just wanted to flag that here.

Reviewer 1

General statement
The authors innovate on classical temporal response function approaches by dynamically time warping the standard N400 shape to modulate its amplitude, latency, and duration. Two contributions stem from it, one methodological, and one neuroscientific. First, the added degrees of freedom improve model predictions of EEG data recorded in passive listening tasks. Second, the approach is used to show that the N400 changes in amplitude and latency depending on word expectedness.

In my view, the study is methodologically sound, and its claims are supported by the analysis results. The manuscript has clear and transparent writing. I have some concerns, mostly small, and a larger one.

Many thanks to the reviewer for the positive comments here.

Larger concern
I feel that the study does not adequately situate itself within the field. In my reading, the contributions are twofold: (1) warping TRFs improves model fits, and (2) N400 latency and amplitude changes as a function of expectancy. Mainly for the second point, but also somewhat for the first, context is missing.

The introduction notes how N400 is ordinarily computed as an average that blurs timing variations. It raises the hypothesis that N400 varies based on lexical context and expectancy. The introduction leaves

the reader with the impression that this is a new hypothesis. Moreover, in the Discussion (line 597 to 602), it is stated that N400 latency effects based on expectancy have not previously been studied.

The manuscript would be enhanced if it cites and evaluates previous work, comparing and contrasting its own results with those. There are papers which test N400 expectancy latency differences, the first two find evidence for such an effect, the last one does not. (There are likely more studies, I only looked briefly.)
https://onlinelibrary.wiley.com/doi/10.1111/j.1469-8986.1995.tb01232.x
https://www.sciencedirect.com/science/article/abs/pii/S1053811912002546
https://www.sciencedirect.com/science/article/abs/pii/S0749596X99926608

We fully agree with the reviewer that we did not adequately situate our study in the context of previous work exploring variations in the latency of the N400. We have extensively rewritten the introduction to better motivate our study in the context of prior literature. In our revised introduction we do not wish to give the impression that a potential relationship between word expectancy and N400 latency represents a new hypothesis. Rather, we want to emphasize that – despite there being several studies that have explored this question in the past – there is a strong sense in the literature that such a relationship does not, in fact, exist. We hope the new framing conveys this idea more clearly and thank the reviewer for raising the issue.

It might also be helpful to contextualize the paper not only within the TRF literature, but also to DTW. Warping has been used before to improve model fits in other EEG contexts:
https://www.cell.com/neuron/fulltext/S0896-6273(19)30894-3?dgcid
https://www.nature.com/articles/s41562-022-01386-8

Again, we thank the reviewer for another helpful suggestion here. We have added some brief discussion of how our framework contrasts with these other DTW approaches – specifically when we first introduce our framework near the beginning of the results section. We do not belabor the point in the manuscript – just because we see these approaches as somewhat different – with ours focused on warping responses to specific stimuli and these other approaches more focused on warping neural data relative to oscillatory cycles or across trials.

Smaller concerns/questions
- How did the authors crop the original and shifted TRF to let them have the same start and end time? (L194).

Apologies for not being clear on this. The static TRF has a fixed convolution window – which we have set in this study to be τ_{min} = 0 ms and τ_{max} = 700 ms. For the dynamic TRF, we allow time shifts of +/-200 ms. Thus, the convolutional window of the dynamic TRF can vary for any individual word up to an interval of -200ms to 900ms. However, in practice, we cropped the time-shifted TRF to let it have a window length of 700ms. In other words, if the analysis were to produce a time shift of -100ms, then the dynamic TRF would now run from -100 ms to +600 ms. Importantly, however, these timings (relative to the word onsets) are conserved when inserting the static and dynamic TRFs into the modeled EEG

timecourse. We have added this information to the methods section – and clarified that we do not believe it to represent any kind of confound.

- Perhaps the visual presentation of the results can be improved by showing more example model fits (beyond Figure 4C). This gives the reader a visual sense of how the various methods and conditions predict the EEG data. I have a suggestion: a 3x2 plot

	Static TRF	DTW TRF	Real data
Low expectancy			
High expectancy			

Each subplot could be populated with the average trace, or if it is not too cluttered, individual traces for all the words. With trace here I mean the predicted or true EEG signal as in Figure 1C.

We appreciate this suggestion and have carefully considered it. However, we are not sure we can think of a better way to display things than we have already done in Figure 4C. Specifically, we do not think it would be helpful to plot individual traces for all the words – there are 11,419 words in our single-speaker stimulus and 9,462 words in our attended cocktail party stimulus – and, as such, a plot of all traces would be unreadable. We are also unsure that dividing the plots into two bins (low and high expectancy) would be better than what we have plotted. What we like about our plot is that it gives the sense of a continuum across expectancy (surprisal) values – and we would prefer to keep this rather than dividing the data into two discrete bins.

- Would it not make more sense to use the average N400 shape in these data rather than the Fourier basis sets? Does the template N400 have an empirical or theoretical grounding that is superior to a data-driven template?

This is a good question. The reason for the use of the Fourier basis sets is practical. Specifically, because there are so many words in our stimulus, we have elected to use an EEG prediction optimization approach to fit our three parameters of interest (amplitude, time-shift, time-scaling). To do this, we chose to incorporate a neural network (really just one convolutional layer) into our analysis framework. To fit the weights of this network, we need to compute errors between the real EEG and our predicted EEG and then backpropagate that error through the network. As is typical with backpropagation, this involves computing gradients so we can "descend" those gradients to minimize the error. As such, we need a TRF that is differentiable. The average N400 shape is a complex waveform that is not directly describable as a differentiable function. This is the reason we had to represent it using a basis set. We hope this makes sense. We have edited our description of the justification for using basis functions in the results section and hope it is clearer now.

Signed,
Sander van Bree

Reviewer 2

This paper proposes that unexpected words delay EEG evoked responses and increase their amplitude. A previous study from the same lab found that the amplitude of the response at around 400 ms increased with surprisal when semantic "dissimilarity" was used as a regressor, resulting in an N400-like response. The novelty of this paper is that more surprising words also delay the response to a greater extent. However, the Discussion section does not contextualize this finding within the existing literature, making it unclear how to interpret it.

Many thanks for taking the time to review our manuscript. We have tried to better contextualize the contribution of our study by extensively editing the introduction section. Specifically, we have sought to emphasize that there is only very limited support in the literature for the idea that variations in language tasks/stimuli can influence the latency of N400 (or N400-like) responses. We suggest a couple of reasons as to why this might be the case and propose that our study adds value by 1) using naturalistic stimuli, and 2) using a data-driven modeling approach that does not require averaging across many stimuli that might vary in subtle ways.

The methods used to extract changes in delay and amplitude with varying surprisals are not necessarily incorrect, but they are unclear and leave room for speculation about the actual procedure. Simpler existing methods, such as the Unfold toolbox or least squares for amplitude modulation, combined with a straightforward search for varying time delays, could have been employed. Using a general-purpose machine learning toolbox may have introduced unnecessary complexity. While this can be rectified, the authors must provide a more detailed explanation of their methodology if they choose to retain it.

Thanks for this comment. It has helped us to appreciate that we need to be clearer about the reasons for the use of our (admittedly complicated) approach. The intuitions behind the reviewer's suggestion of using the Unfold toolbox and searching for time delays is very much the same as that behind our approach. Indeed, the mTRF toolbox and the Unfold toolbox are very similar in their goals of deconvolving neural responses to stimuli that occur very close in time (or even continuously). They are also very similar in their implementation. [For what it's worth, the mTRF toolbox works very well for deconvolving responses to discrete stimuli that are close in time, it is not restricted to only continuous stimuli.] The reason we have chosen to use our approach centers mostly on the fact that there are 11,419 words in our single-speaker stimulus and either 5,097 or 4,365 words in our attended cocktail party stimulus, depending on which story a subject was attending to. Doing a brute force search across delays for that many words would have been intractable. So, our goal, was to do such a search in the context of a framework where we aimed to optimize how well we could model (i.e., predict) the EEG responses to our stimuli. By framing the problem as an optimization problem, we could leverage the tools of machine learning (i.e., gradient descent and backpropagation) to fit parameters that would describe the time-shift (and amplitude change) as a function of the word suprisal. The use of the mTRF toolbox vs the Unfold toolbox was merely a matter of preference given that that is what our lab is accustomed to. But, as mentioned above, the two toolboxes essentially do the same thing. So, again, the reviewer's intuitions and ours are the same here. Our use of a machine learning style approach was

really just a question of tractability. We have added some text to the early part of the result section to make this point more explicit.

The primary concern is the lack of a clear connection between the Python/machine-learning description of the process, the model equations, and the model diagram.

We address this issue in various ways, which we describe in response to some of the specific comments below. Overall, we are hoping that our efforts to explain the scale of the challenge in terms of modeling responses to ~10,000 words and our choice to formulate this as an optimization problem – will serve to make this issue clearer.

So in total, it is not clear that the results are new, nor is it clear that the methods are of wider interest.

We hope that our new introduction does a better job of making the contribution of the study clearer in terms of the debate over the fixed nature of N400 latencies. We would also like to note that the use of encoding models to describe neural responses to natural speech continuous to grow in popularity. As such, describing methods that can improve the prediction accuracy of such models represents another contribution that should have value. This is something we have also tried to add more emphasis on in the revised manuscript.

More detailed comments:

Paragraph of line 219 is cryptic. I doubt it can be made much easier to understand, because the actual approach seems clunky. I doubt you will be inclined to change the approach (though that is what I would recommend). So you have no other option but to explain what you did in a way that is actually comprehensible. I tell you what I understand, and you fill in the blanks please. You fit the TRF in the time domain, rather than the time domain. Fine. Then you adjust, for each trial, amplitude, time delay and scale (shared for all EEG channels) to better fit individual trials. Why this could not have been done more simply in the time domain is not clear to me. What any of this has to do with "linear convolutional layer" and "backpropagation" I have no idea. The least-squares solution to the Fourier (or time domain) coefficients can be solved in closed form. Fitting after that the three extra parameters per trial with non-linear least squares should not be a big deal. Admittedly, adding delay and time-scales makes this non-convex nonlinear optimization, but you have a good starting point so non-convexity should not be a problem.

The reviewer's thoughts here very much align with our actual approach. We apologize that the way we wrote things may have been confusing. We think the confusion centers on the reviewer's concern that "*You fit the TRF in the [frequency] domain, rather than the time domain.*" However, this is not correct. Our entire analysis is based in the time domain. However, as discussed above, we have chosen to do this via a machine learning approach based on the fact that our stimuli – being narrative audiobooks – have 11,419 words and 5,097 or 4,365 words. As such, performing a brute force search for an optimal time lag for each individual word would be intractable. [As a note here: we are not quite sure what the reviewer means by "trials". Our trials are lengthy (1-3 mins) segments of audiobooks. And our analysis is

focused on every individual word across all of our trials.] To get around this issue, we have opted to cast the problem as one of optimization – in other words, we have set up a framework where we aim to optimize how well we can predict EEG responses based on the surprisal of the words in our story. This allows us to compute an error between the EEG we predict and the actual, true EEG. Then we can use machine learning methods to backpropagate that error through a neural network (in our case just a single convolutional layer) to learn the weights of the three parameters of interest. To be able to do this, the static TRF needs to be represented as (a collection of) continuous functions – because backpropagation works by performing differentiation. This is what we mean by Fourier basis functions. They are not frequency domain components, but sinusoidal basis functions (in the time domain). As such – and just in line with the reviewer's intuitions – our approach does indeed learn time-domain, word-by-word adjustments. It just does so via (what are these days fairly standard) machine learning methods to make it tractable given that our stimuli contain so many words (11,419 or 5,097 or 4,365). We have reworked the early part of the results section significantly to make it clearer why we chose this approach and to more clearly explain the necessity of re-representing the TRF as continuous basis functions.

Maybe using ready-made python code "scikit-fda library" made the description unclear?

Apologies – we have added some explanatory text to make clear that this library enables one to re-represent data in terms of continuous functions – such that those functions can be differentiated so that one can perform backpropagation to optimize one's parameters.

To gain clarity, I went to the methods (Eq. 7-15). They add little clarity. What are s_i and M_i? In Fig 1 N is the length of the convolution window and s_i are apparently the regressors. The Equations 9-15 make no sense to me.

Sincere apologies – the reviewer's comment has made it clear that we had not included all of the necessary information in this section. We have substantially revised it – parsing it in a way that should make it easier to follow – and adding in more detail on the meaning of the specific parameters and how those parameters are fit.

Trying again to understand, it appears from Fig. 6 that you allow the amplitude a_i and time delay b_i to scale linearly with surprisal of the current and the previous word. Why? That really makes the interpretation of results complicated. The coefficients seem to be negative for the s_{i-1} so it looks like amplitude scales with surprisal change. Do you feel strongly about that? Then say it in the abstract.

One hypothesis we had was that the brain's response to the current word might depend on the surprisal of that word – but also on the surprisal of the previous word. The intuition here is that, if one hears a surprising word, one might be less surprised if the next word is also something unpredictable. This turned out to be the case. The negative coefficient for the previous word means that if the previous word is highly surprising, it reduces the effect of the current's word surprisal on the TRF shift. We mentioned in the abstract that our "effect is driven both by the word's own predictability and the predictability of the immediately preceding word". But we are slightly reluctant to add any more detail

to the abstract for fear that it would make it overly complex. We do discuss the interpretation on page 23.

You may also consider using the Unfold toolbox. While it is NIH (not invented here) we have used it and it really works nicely to get the different TRF (shapes) at varying amplitudes of the regressor. It has been well validated and has a clean rationale. It does give you maybe too many degrees of freedom, so you will not be able to use it single-trial, but you can at least bin into different amplitudes of the regressor making the kinds of statements you want to make. Maybe it will work for you.

Thanks for this suggestion. Once again, the reviewer's intuition here aligns very much with our own. The mTRF toolbox and Unfold toolbox essentially do the same thing (deconvolve overlapping responses). Our choice here was motivated by the number of words in our study (11,419 words and 5,097 or 4,365 words) and our own extensive experience with the mTRF toolbox which made it a more natural choice for us. Using the mTRF toolbox also made it somewhat easier to quantify how well our dynamic TRF could do at predicting unseen EEG (i.e., it not only allowed us to show the effects of surprisal on the TRF/ERP – but it also allowed us to quantify the model improvements).

The main question is if this paper is about a new method, then the methods need to be explained with much greater level of detail. If the paper is about the empirical result, it may be better to use simpler methods that make the same case without distractions.

Our manuscript had three main goals. Two of them were focused on empirical questions: 1) does word surprisal influence N400 response latency; and 2) does the identification of a word rely on the interaction between the word's predictability and the word's phonetic structure. As discussed above, answering these empirical questions required us to use a slightly more complicated method given the number of words in our stimulus set. This method also served to achieve the third goal – which was to provide the community with a way to improve the modeling of brain responses to natural speech – something that, as discussed above, is becoming more popular in recent years.

As to results, Fig. 2, I am also confused. Of course adding single-trial parameters will improve Pearson's r. You would have to demonstrate this on unseen data. But you can't, as they have been fit to every trial. So some sort of shuffle argument has to be made that the observed improvement in r is larger than what is expected by chance with the extra single-trial parameters. The Wilcoxon test across subjects misses this point. Unless I am really not understanding something here. So I am not sure what to make of panels A and B.

The reviewer makes an important point here indeed. We regularly deal with the issue of model comparisons where one model may have more parameters than another. And, in line with the reviewer's idea, we often carry out control analyses using shuffled data to validate any putative model improvements. In the present study, this is complicated by the computational complexity of our analysis. Running our analysis through numerous shuffled datasets would take an incredibly long time. However, just like the reviewer, we were concerned that the results from Figs 2A and B could arise as a

result of overfitting. A first thing to say on this is that we did not actually fit our models on all our data (again, we think there may be some confusion over the meaning of the word “trial” here; we use it to refer to the minutes-long audiobook trials, but the analysis focuses on responses to individual words). Rather, we use cross validation to train our static and dynamic TRFs on some trials and test on the left-out trials. This is described in the “Cross Validation” section in the Methods. Secondly, concern over potential overfitting was also the primary reason for our inclusion of the second data set in the study – namely, the cocktail party dataset. Our reasoning was that – if our results are simply driven by overfitting – then we should see model improvements for both attended and unattended speech. However, if our results are driven by real changes in neural responses to words as a function of their expectancy, then that should only hold for attended speech. This turned out to be the case – Fig 3 – and is something we discuss in some detail in the manuscript. We would like to make two additional comments on this important issue. First, it turned out that not all added features improved the modeling. As discussed in the manuscript, the time-scale feature did not lead to any improvement. This give some additional support to the idea that the other features are capturing something real. And second, even more support for the notion that the other features are capturing something real comes from the demonstrable changes in we see in the TRFs (Fig 4). These changes are both interpretable (more surprising words may take longer to process) and they explain why the prediction accuracy is improved (because accounting for variations in the response profile as a function of surprisal improves the ability to model EEG responses to speech). We think these factors – no improvement for unattended speech, improvement for some features, but not others, and the interpretable influence of parameter changes on the response – collectively provide strong support for meaningful improvements in model accuracy that cannot simply be explained based on extra parameters. We have added more details on this important issue to the relevant paragraph in the discussion.

I was also confused about the lines in panel C, and only a day later did I realize that this may be the linear weights combining s_i an s_{i-1}. So we have:

a_i = beta_0 * s_i + beta_{-1} * s_{i-1} ?

This is correct – in the text in the revised manuscript we now explicitly link the results in this figure to the variables in the methods section.

If so, this can be solved in closed-form, no need for gradient descent. But maybe not, because of Fig. 4, where it seems the entire shape of the filter H (TRF) changes with surprisal.

We apologize but are not sure we fully understand this comment. As discussed above, our analysis aims to better capture EEG responses to many thousands of words. It is not clear how we would solve for the relevant time-shifts and amplitude changes in closed form.

Figure 4 B, makes sense and is interesting. A much much simpler way to show this would be to do a TRF for low and high surprisals words and show the two curves. It would tell the same message and be

easier to understand. In fact, is that what Fig 4C is? If all that was done was to change amplitude and time shift, why does the entire shape of the functions change? Again, I must not be understanding something basic. By the way, that arrow is really not great. A color bar for surprisals would have been a better choice.

The reviewer is absolutely correct that computing a TRF for low and high surprisal words would largely give the same message (and, of course, be simpler). However, it is also true to say that it would be decidedly suboptimal. One of the key messages of our manuscript is that responses vary in their amplitude and timing over a continuum of surprisal levels (e.g., Fig 4B, Fig 4C). Binning this variation into two arbitrary bins would fail to exploit the full variation in the data. And, as such, it would lead to EEG prediction accuracies that would be significantly worse than those we report here. Given that our manuscript aims to address some empirical questions AND to contribute some new methodological ideas (enhancing standard linear regression modeling), we decided to take our present approach. We have discussed this choice – and the alternatives – near the beginning of the result section. On the issue of the arrow, we quite like it and would prefer to keep it please.

On the issue of "uniqueness point", it seems clear that you want to separate that form surprisal. What is the "uniqueness" point exactly. Surprisal is coded both as a point in time and and amplitude, i.e. a pulse at word-onset corresponding to the level of surprisal. What exactly is "uniqueness point"? Also a time and an amplitude? Or just a unit-amplitude pulse that the time point where phoneme entropy no longer drops within a word?

Apologies for the lack of clarity on this issue. The reviewer's question is a good one. We initially considered placing impulses at the time point where phoneme entropy no longer dropped. But we realized it was practically far easier – and just as valid – to place impulses at the start of each word whose amplitudes defined the uniqueness point of the word, i.e., words with later uniqueness points have higher amplitude impulses at word onset. The reason this was practically easier is that it more naturally fit into the framework we had developed for surprisal. The reason it is just as valid is that – if there is indeed a difference in TRF shape for words with earlier vs later uniqueness points, then the framework should warp the TRF in the same way as it did for surprisal. The reality was that this did not really happen. Although we were able to confirm that the uniqueness point did not drive the surprisal effect we report. We have added a footnote to clarify how we implemented the uniqueness point analysis.

Incidentally, should surprisal not be coded at the word offset, rather then the word-onset? How would that impact the timing results?

This is a great question! We have thought about this quite a lot. Indeed, this was the main reason why we thought to do an analysis involving the uniqueness point. In line with the reviewer's intuition, our idea was that people's brain responses to word meaning should be locked to the timepoint at which they recognize that word. As such, we thought including information about the uniqueness point (the time at which they should be able to fully recognize a word – they shouldn't necessarily need to wait to the very end) would be informative. But it turns out it was not – which is a bit surprising but also

interesting. I guess we shouldn't have found it too surprising given that the long history of the N400 is all based on an analysis that locks to word onset! And, according to most of the previous literature, the N400 latency has not been particularly sensitive to different words/tasks!

Minor comments:

Previously you argued that N400-like TRF is due to semantic dissimilarity. Now it is explained in terms of lexical surprisal. Same or different? Seems like worth a Discussion.

Thanks for bringing this up. They are indeed different – at least in terms of how they are calculated – although they certainly tap into similar underlying neural activity. Our original use of semantic dissimilarity was largely due to the fact that it was something we could compute using models that we had access to at the time (Word2Vec). With the arrival of transformer models like GPT (which are trained on next word prediction – well, really next token prediction), we have switched to word surprisal. There are two main reasons for this. First – it is, frankly, not quite clear what semantic dissimilarity means. It is also a somewhat tricky measure to accurately compute given that older models like word2vec do not take account of context when determining a word's "meaning". And, second, quantifying words in terms of how predictable they are based on their preceding context – which we do now – is much more analogous to the classic N400, which – as the reviewer is undoubtedly aware – is based on the word's predictability according to cloze probability. We have added some brief discussion on this issue as a footnote when we first introduce the notion of fitting TRFs to lexical surprisal.

line 32: "only varies (linearly) in terms of its amplitude" linear with what? Strength of a feature? Predictability? Dissimilarity? I happen to think that most EEG amplitude correlates reasonably with prediction error (MMN, ERN, P300, N400, etc). Amplitude may not scale linearly, that is clear. The "Unfold" toolbox does a nice job with that.

This is a reference to the previous sentence where we describe how previous work has fit encoding models to describe how semantic dissimilarity or surprisal relates to EEG responses. The point we are trying to make is that previous encoding model work has fit *linear* models between speech/language features and EEG responses – whatever those speech features happen to be. Such linear models assume that when the speech feature doubles in size (for example, when one word is twice as unpredictable as another), then the resulting response has the same shape and timing, but is twice as big. This is analogous to saying that an N400 to a very unpredictable word is bigger than one to a less predictable word. Or that a P300 to a rare oddball is larger than one to a less rare oddball. Or that a P100 to a high contrast checkerboard is larger than that to a low contrast checkerboard. But it is within the framework of a model that casts the ongoing EEG a series of temporally overlapping responses to events that occur in close proximity (or that could even be continuous – e.g., the speech envelope). The key point of the sentence is to convey that amplitude responses may not scale linearly – but also that the shape might

not necessarily stay the same for every level of a features (i.e., high predictability or low predictability) and that the goal of our study is to relax that constraint.

Line 36: “expectedness” … hmm, you already introduced “semantic dissimilarity” last time around, now expectedness. Everyone is allowed their own words. But do we really have to introduce a new one every time? We already have “surprise”, “novelty”, “perplexity”. Those are all fine words one may want to use. You also used “surprisal” (e.g. figure 2) which seems more common in this literature. Do we really need “ expectedness” now? Since your argument seems to be about prediction, maybe say simply “prediction error”?

A fair point indeed – the draft went through multiple iterations – this was a holdover from an early draft. We have chosen to replace “expectedness” with “predictability”.

And actually you call it “lexical surprisals” in most places. My understanding is that this is usually defined as the neg log of word frequency in the language. Whereas you use a 1024 word context and a fancy language model, so really it is the next-word likelihood given prior context. Maybe “semantic surprisal” or “language model surprisal” would be a better term? Lexical surprisal will be confusing to most readers.

Lexical surprisal would really only be the negative log of word frequency if one was listening to random word lists. When words appear in a context their predictability (and hence their surprisal) obviously varies. What we are dealing with in this study is indeed context-based lexical surprisal. Although that phrase is a bit clunky to use throughout the manuscript. And semantic surprisal is not quite accurate – it is not entirely clear how much the predictions from these language models are driven by semantics. Also, language model surprisal seems a little awkward to us. What we have done in response to the reviewer’s helpful comment is to define what we mean by “lexical surprisal” right at the outset and to make sure that it is clear how that differs from lexical surprisal as relating to word frequency.

Line 564: “Time&Amp dynamic TRF”. I think you can afford the extra charters to write out these words.

We understand the reviewer’s point here indeed. However, we actually felt that this short naming convention might be more helpful than harmful in keeping confusion to a minimum. We wanted to make it clear that there were different parameters that could through which the TRF could be modified and that this particular combination – the Time&Amp version – was the one that added value. We are inclined to stick with it please.

Line 565 “dynamically warped TRF”, you end up just shifting and scaling amplitude. Maybe “warp” is not the right word.

Thanks for this. We have simplified things and just refer to it now as the dynamic TRF.
