# Supplementary material for: Dynamic modeling of EEG responses to natural speech reveals earlier processing of predictable words
Source: PLoS Comput Biol. 2025 Apr 28;21(4):e1013006. doi: 10.1371/journal.pcbi.1013006 (PMC12061398; doi:10.1371/journal.pcbi.1013006)
Supplement: Attachment — Submitted filename: Review.docx [file pcbi.1013006.s005.docx]

**PCOMPBIOL-D-24-01459**

**Dynamic modeling of EEG responses to natural speech reveals earlier processing of predictable words**

**General statement**

The authors innovate on classical temporal response function approaches by dynamically time warping the standard N400 shape to modulate its amplitude, latency, and duration. Two contributions stem from it, one methodological, and one neuroscientific. First, the added degrees of freedom improve model predictions of EEG data recorded in passive listening tasks. Second, the approach is used to show that the N400 changes in amplitude and latency depending on word expectedness.

In my view, the study is methodologically sound, and its claims are supported by the analysis results. The manuscript has clear and transparent writing. I have some concerns, mostly small, and a larger one.

**Larger concern**

I feel that the study does not adequately situate itself within the field. In my reading, the contributions are twofold: (1) warping TRFs improves model fits, and (2) N400 latency and amplitude changes as a function of expectancy. Mainly for the second point, but also somewhat for the first, context is missing.

The introduction notes how N400 is ordinarily computed as an average that blurs timing variations. It raises the hypothesis that N400 varies based on lexical context and expectancy. The introduction leaves the reader with the impression that this is a new hypothesis. Moreover, in the Discussion (line 597 to 602), it is stated that N400 latency effects based on expectancy have not previously been studied.

The manuscript would be enhanced if it cites and evaluates previous work, comparing and contrasting its own results with those. There are papers which test N400 expectancy latency differences, the first two find evidence for such an effect, the last one does not. (There are likely more studies, I only looked briefly.)

<https://onlinelibrary.wiley.com/doi/10.1111/j.1469-8986.1995.tb01232.x>

<https://www.sciencedirect.com/science/article/abs/pii/S1053811912002546>

<https://www.sciencedirect.com/science/article/abs/pii/S0749596X99926608>

It might also be helpful to contextualize the paper not only within the TRF literature, but also to DTW. Warping has been used before to improve model fits in other EEG contexts:

<https://www.cell.com/neuron/fulltext/S0896-6273(19)30894-3?dgcid>

<https://www.nature.com/articles/s41562-022-01386-8>

**Smaller concerns/questions**

- How did the authors crop the original and shifted TRF to let them have the same start and end time? (L194).

- Perhaps the visual presentation of the results can be improved by showing more example model fits (beyond Figure 4C). This gives the reader a visual sense of how the various methods and conditions predict the EEG data. I have a suggestion: a 3x2 plot

Static TRF	DTW TRF	Real data

Low expectancy

High expectancy

Each subplot could be populated with the average trace, or if it is not too cluttered, individual traces for all the words. With trace here I mean the predicted or true EEG signal as in Figure 1C.

- Would it not make more sense to use the average N400 shape in these data rather than the Fourier basis sets? Does the template N400 have an empirical or theoretical grounding that is superior to a data-driven template?

Signed,

Sander van Bree
