# Supplementary material for: Dynamic modeling of EEG responses to natural speech reveals earlier processing of predictable words
Source: PLoS Comput Biol. 2025 Apr 28;21(4):e1013006. doi: 10.1371/journal.pcbi.1013006 (PMC12061398; doi:10.1371/journal.pcbi.1013006)
Supplement: Attachment — Submitted filename: Response to Reviewers.docx [file pcbi.1013006.s008.docx]

Many thanks once again to the reviewers for their time and efforts in commenting on our manuscript. We have addressed those comments and think that our manuscript has been further improved as a result. We respond to each comment below.

Reviewer #1: Thank you for these revisions. The paper now better integrates previous work on the variability and timing effects of N400,and I thank the reviewers for responding to my concerns. My last issue is that the new introduction is written in an eccentric style that lacksclarity and structure. I think the Author Summary is quite good, and the paper would be enhanced if the introduction could adopt thatsame level of sharpness. I would not appeal to intuitions ("surprising", "one might expect") or rely on review/expert quotes and judgments.The writing and presentation of results could be more matter-of-fact, focusing on empirical results, theoretical arguments -- in a nutshell,more objective. The use of footnotes further suggests the writing is not as streamlined as it could be. One variation:

The overarching question is how humans process speech, N400 is a phenomenon that robustly shows up, here are experiments that showit is static (concisely describe), here are some results it is dynamic (concisely describe). Here are is why the dynamic picture may be moreplausible (using frameworks, theoretical reasons, arguments, other results), here is what we do to sort out the debate, etc.

Thanks to the reviewer for this. We agree that it is important to be more objective and clearer in the framing of our study. We have removed the appeals to intuition and rewritten part of the introduction in a manner more in line with the reviewer’s suggestion. We have also moved part of the introduction to the discussion where it (now) fits better.

In terms of the footnotes – we do not usually use footnotes – but, in this case, we quite like the idea of keeping them. The first footnote relates to a change in how our lab (and others) quantify the “amount of meaning” a word adds to its context. Previously, we did this using semantic dissimilarity. But, as language models have moved on, we have switched to using lexical surprisal. We have preprinted some work on this since our original semantic dissimilarity paper. We think it would distract somewhat from the point of the present paper to discuss this in too much detail in the main text. But we agree with Reviewer 2 that readers may not be familiar with our preprint and that it is worth explaining the switch in measure. We thought this was a good case for a footnote. The alternative might be to refer to some supplementary materials and include the paragraph there. We thought a footnote was a bit more expedient for both us and future readers. The second footnote relates to how we implemented the uniqueness point analysis. We describe the method in the main text. However, we (and we suspect future readers) might find that somewhat unintuitive as there is another (likely more obvious) way to do it. So we just wanted to explain why we chose the method we chose and that it is – essentially – equivalent to the more obvious way. Once again, we thought a footnote was a nice way to do this so as not to overload readers – but also to provide interested readers with that extra information.

All that is to say – we would rather keep the footnotes in this case.

Reviewer #2

What is the j in r_{ij}? I don’t see how that is ever summed over? But maybe one should not, because as you write in line 925, j stands for index of a “channel”. Do you mean EEG channel? But then, why is the j with the basis function in equation 8. Should it not be d_{ij}? Or do you really mean that say that every EEG channel uses its own set of basis functions?

And why not match the notation of equation 8 with Fig. 1A to help your readers understand?

Thanks very much to the reviewer for their attention to detail – we should have been more careful ourselves in explaining things – apologies!

We have now aligned equation 8 with Figure 1, and we have clarified the channel is TRF channel. We have also updated figure 1A to add channel subscripts to h and d. We also removed the $h\left( \tau\right)$ from the static TRF equation in the Figure 1C, because static TRF is just $h[\tau]$, the discrete version.

We also changed the naming of warped TRF to dynamic TRF in Figure 1 and Figure 4.

And we added the multiplication operator to the equation 6.

Figure 6 is unchanged and remains cryptic to me. The equations talk about a scale parameter c_i but it does not show in the figure. Sowhich is it? Do you, or do you not use a variable time scale for each word? And if you did not vary time scale, maybe that should not be in the abstract either?

We have added a time scaling node to Figure 6. We mentioned that we tried time scaling but it doesn't provide additional benefits.

By the way, for the longest time c(t-t_i) looked to me like c() is a function of time, when really it is a scalar.

Now that I understand the notion of sinusoidal basis functions, I worry about non-convex cost for the c and b parameters that are in theargument of the sin/cos functions. I guess you are fine with a local minimum? Might be worth discussing the initial conditions in these circumstances. I suppose you initialize so that c=1, b=0?

We didn't initialize the values of c and b because they are middle output of the convolution layer. We follow the default initialization of how convolution layer was initialized in pyTorch. But we do set the default value of a, b, c when we don't enable the fitting of them. We clarified these values in the manuscript at line 940.

On the other hand, a_i seems linear with s_i, and the proportionality coefficients beta should be solvable in closed form. Roughly, for givenh_i(t) = h(t-t_i):

beta = argmin_beta sum_t(eeg(t) – beta * sum_i s_i*h_i(t))^2 = eeg\(s*h)

But I guess, once you decide to use gradient descent, might as well do it for the quadratic term as well. Makes sense.

Regarding using gradient descent, one point is that it is true that we can derive the weight for the a_i in a closed form. But it is not as easy when we also include time-shifting and time-scaling parameters and solve them together. Another point is that we hope in the future with more data, we can replace this linear module with non-linear modules that can capture more complex relationships between lexical surprisal and transformation parameters.

By the way, you still talk about “warped” in several places in your text. “warped lexical suprisal” is a particularly interesting wording. :-)

Thanks for catching this. We have now removed all instances of the word warped.
